# Body fat assessment in youth with overweight or obesity by an automated bioelectrical impedance analysis device, in comparison with the dual-energy x-ray absorptiometry: a cross sectional study

**DOI:** 10.1186/s12902-022-01111-6

**Published:** 2022-08-02

**Authors:** Hanen Samouda, Jérémie Langlet

**Affiliations:** 1 Precision Health Department, Luxembourg Institute of Health, Strassen, Luxembourg; 2 Business Development Office, Luxembourg Institute of Health, Strassen, Luxembourg

**Keywords:** Adiposity, Body mass index, Overweight, Obesity, Fat mass, Dual-energy x-ray absorptiometry, Bioimpedance

## Abstract

**Background:**

Bioelectrical impedance analysis (BIA) is a widely used method to assess total body fat (TBF) depots characterising obesity. Automated BIA devices provide an inexpensive and easy assessment of TBF, making them widely available to the general public and healthcare providers without specific qualification to assess body composition. The equations included in the automated BIA devices have been developed in very few specific populations, which means that they are not suitable to assess TBF for everyone and need to be validated before use in other populations. **The aim** of the present work is to evaluate the accuracy of the automated BIA device Tanita® BC-532 in youth of White European ethnicity, compared with the dual-energy x-ray absorptiometry (DEXA), gold standard measurement of TBF.

**Methods:**

Total body fat percentage (TBF%) was measured with the BIA device Tanita® BC-532 and DEXA (Hologic® QDR4500W) in 197 youth of White European ethnicity (*N =* 104 girls), 7-17 years old, and visiting the *Diabetes & Endocrinology Care Paediatrics Clinic, Centre Hospitalier de Luxembourg,* for overweight or obesity management.

**Results:**

TBF% evaluated with BIA was significantly correlated with TBF% measured with DEXA in both boys (r _Pearso*n*_ *=* 0.617) and girls (r _Pearso*n*_ *=* 0.648) (*p* <  10^− 4^). However, the residual mean between the assessment of TBF% by BIA and by DEXA [TBF _BIA_ (%)-TBF _DEXA_ (%)] is extremely high (mean ± standard deviation = 10.52% ± 5.22% in boys, respectively 9.96% ± 4.40% in girls). The maximal absolute residual value is also very high, about 24% in both genders.

**Conclusions:**

The automated BIA device Tanita® BC-532 appears to be not accurate to assess total body fat in youth with overweight or obesity. There is a need to calibrate the BIA device before its use in the populations where it was not previously validated.

## Background

**Body mass index (BMI)** assessment has been widely used to define or diagnose obesity [1].

BMI values of more than 30 kg/m^2^ have been recommended by the World Health Organisation (WHO) in order to diagnose obesity [1]. This cut-off has been commonly used worldwide, yet widely criticised for its poor sensitivity or ability to identify people having high total body fat (TBF), which in fine, defines obesity [1, 2]. A systematic review and meta-analysis showed that a BMI threshold ≥30 kg/m^2^ fails to detect more than half of individuals with a high total fat mass due to a sensitivity value of the BMI not higher than 0.42, in comparison with the reference measurement of body fat by Dual-Energy-X-ray-Absorptiometry [3]. Actually, BMI does not distinguish between fat mass and fat free mass at the individual level. For a same high value of BMI, body composition might considerably vary between individuals. People having a high muscle mass or bone mass might be wrongly considered as having overweight or obesity [4–8].

**Dual-Energy-X-ray-Absorptiometry (DEXA)** is currently considered as the reference method for body composition assessment, providing a gold standard measurement of the body fat mass, muscle mass and bone mass at both total and regional levels (trunk, arms and legs) [9–13]. With around *3%* margin of error, DEXA provides a highly accurate measurement of the different body compartments [9–13]. Body composition measurement by DEXA is easy to perform, rapid, with very low radiation exposure and very little inconvenience for the patient. These characteristics promoted DEXA use in clinical practice and research in order to accurately assess body fat [9–13]. Nevertheless, despite its multiple advantages, the major disadvantage of DEXA resides in the fact that the analysis requires an expensive equipment that few clinicians have at their disposal for a daily basis usage. DEXA equipment is also almost never available for a regular usage by researchers. Moreover, DEXA measurements of body composition cannot be performed in population based studies [9–13].

***Bioelectrical impedance*** **analysis (BIA)** is an indirect assessment method of TBF, widely used in research and clinics. The method is based on the capacity of the hydrated tissues in the body to conduct electric current. The BIA devices include predictive equations of body fat developed in specific populations. These BIA devices measure the electric bioimpedance through Ohm’s law, when a sinusoidal electric current of low intensity (± 1 mA, mA) and at high frequency (more than 50 kHz, kHz) is transferred between several predefined points on the body through voltage [14]. The bioimpedance measures the resistance of the biological tissues to the electric current conduction. The equations included in the devices provide a calculation of the intra-cellular and extra-cellular fluids, and the total body water using the measured electric impedance and resistance for each segment of the body, and predict total fat mass and fat free mass (FFM) [15–17].

**Automated BIA devices:** More recently, less expensive and highly simplified BIA devices for TBF assessment have been developed. These BIA consist in bipedal, bimanual, or a combination of both devices [18–22]. These devices contain integrated electrodes inside, sending small electric current through the total body water. Electrodes positioning is therefore made easier and the body fat measurement faster. Bipedal BIA channel the electric current from foot to foot. In bimanual devices, electric current circulates from hand to hand. TBF is calculated based on the measured impedance [18–22]. These BIA analysers are portable, automated and easy to use, provide a direct reading of TBF and are widely available in the marketplace at affordable prices. This therefore highly increased their usage in non-specialising clinical setting and amongst the general public. However, automated TFM calculation provided by these BIA devices is based on nonspecific predictive equations, pre-established by the manufacturer and different according to the device, which raises the question of its accuracy [23]. In addition, body composition varies according to the ethnicity and environmental exposure [24–26]. Furthermore, the equations included in the automated BIA devices have been developed in very few specific populations, which means that they are not suitable to assess TBF for everyone and need to be validated before use in other populations [27–30].

**The study aims** to evaluate the accuracy of the automated BIA device Tanita® BC-532 in youth of White European ethnicity, in comparison with the DEXA.

## Methods

### Study participants

**Fig. 1:**
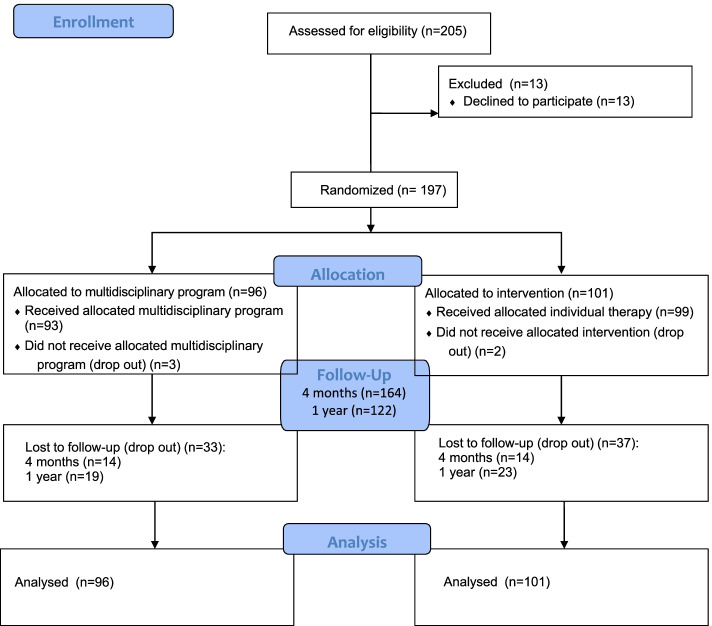
CONSORT Flow Diagram

*N* = 197 youth of White European ethnicity (93 boys, 104 girls) were invited to participate in the study as previously described [31–34]. They were aged between 7 and 17 years old, with overweight or obesity according to the IOTF definition [35], and visiting the *Diabetes & Endocrinology Care Paediatrics Clinic, Centre Hospitalier de Luxembourg* between September 2006 and June 2008 for overweight or obesity management [31–34]. The sample was compiled by inviting all the 7 to 17 years old children and adolescents, frequenting the Paediatrics Clinic and seeking for obesity treatment between September 2006 and June 2008, to participate in the study. Only the youth who had conditions in relation with body composition alterations, including the hypoparathyroidism, a leptin deficiency, the Laurence Moon Biedl syndrome and the Prader Willi syndrome were excluded from the study. The girls took part to the study outside their menstrual period. The data of the present work were collected as part of a study on the impact of a multidisciplinary obesity management group program on health outcomes in children and adolescents with overweight and obesity. The participants were randomly assigned to either the multidisciplinary group program (*n =* 92) or the individual therapy (*n =* 99), according to the gender, age and overweight or obesity status as described in the Fig. 1 (CONSORT flow diagram). Five participants left the study after their allocation into the therapeutic program.

### Anthropometry and physical examination

Height and weight were assessed according with the Lohman’s anthropometric reference manual [36]. The body mass index (BMI) was calculated. The Dutch L, M, and S values were used to define the BMI Z Scores [37]. The free LMS Growth software was used to establish the overweight and obesity cutoffs [38]: the 91th (boys) and 89th (girls) percentiles for overweight, respectively the 99th (boys) and 98th (girls) percentiles for obesity [37, 38]. The pubertal or Tanner stages were defined by physical examination [39, 40].

### Body fat measurement by dual-energy x-ray absorptiometry

The gold standard measurement of total body fat percentage (TBF _DEXA_%) was measured with the dual-energy X-ray absorptiometry using the Hologic® QDR4500W densitometer (Hologic Inc., Waltham, MA, USA). The TBF% was assessed at five conventional predefined areas: trunk, left and right arms, and left and right legs [32, 41].

### Body fat evaluation by bioelectrical impedance analysis

Total body fat percentage was measured with the automated BIA device Tanita® BC-532 (Tanita Corp., Tokyo, Japan) (TBF% _BIA Tanita® BC-532_) according to the recommendations of the manufacturer. The evaluation of the TBF% with the BIA Tanita® BC-532 was performed immediately prior to the DEXA examination. The Tanita® BC-532 is a foot-to-foot bioelectrical impedance device. In order to control the hydration status, the study participants were asked to not eat or drink liquids, including coffee and alcohol for the adolescents, and to not practice vigorous physical activity 8 hours before the measurement. The study participants were standing on a platform scale including electrodes, enabling the electric current to pass from one foot to another. Before the measurement, the study participants provided information on their physical activity level (inactive, active or athlete). This information was entered into the BIA device, in addition to the age and sex information. The impedance is measured via this process and the body fat percentage is calculated through pre-established body fat predictive algorithms included in the device, and taking into consideration the age, gender, weight, height and level of physical activity.

### Statistics

The Kolmogorov–Smirnov test and Q–Q plots were performed in order to evaluate the normal distribution of the data. The accuracy of the Tanita® BC-532 to predict the TBF% was assessed by means of several statistical analyses:

- Student’s t-tests for paired samples to compare the average percentage of total body fat assessed with the Tanita® BC-532 [TBF _BIA_ (%)] and its reference measurement with the DEXA [TBF _DEXA_ (%)],
- Analysis of variance (ANOVA) to assess the differences among the means of the total body fat percentage observed with the _BIA Tanita® BC-532%_ in the 5 Tanner stages,
- Bivariate regression analyses to analyse the associations between the percentage of total body fat assessed by the automated bioelectrical impedancemeter [TBF _BIA Tanita® BC-532_ (%)] and measured by the dual-energy x-ray absorptiometry [TBF _DEXA_ (%)],
- Analysis of the residual values, or differences between the prediction of the TBF% with the BIA Tanita® BC-532 and measurement by DEXA [TBF _BIA Tanita® BC-532_ (%)-TBF _DEXA_ (%)].
- Assessment of the accuracy of TBF% predicted by the Tanita® BC-532 by means of the Bland and Altman [42] adapted representations of the differences between TBF _BIA Tanita® BC-532_ (%) values and the gold standard measurement of total body fat by DEXA [TBF _DEXA_ (%)] in function of the average of the two methods.

The results were displayed as mean ± standard deviation, minimal and maximal values. The *p*-values < 0.05 were considered as significant. We used the SPSS for Windows, Version 25.0 in order to perform the statistical analyses.

## Results

The Table 1 shows the characteristics of the study participants. The reference values of TBF_DEXA_% are about 44.4% ± 6.5% (min: 28.4%; max: 59.7%) in boys, respectively 47.5% ± 6.7% (min: 29.6%; max: 63.1%) in girls. The values of TBF _DEXA_% were significantly different than the values of TBF _BIA Tanita® BC-532_% in both boys [34.5% ± 7.7% (min: 23.4%; max: 58%)] and girls [38.1% ± 6.6% (min: 26%; max: 59.2%)] (*p* <  10^− 4^).

**Table 1:** Study participant’s characteristics

	Boys (***N =*** 93)		Girls (***N =*** 104)	
	Mean ± SD	Min - Max	Mean ± SD	Min - Max
**Age (years)**	11.8 ± 2.3	7.3 - 16.7	12.1 ± 2.4	7.4 - 17.3
**Height (m)**	1.54 ± 0.13	1.27 - 1.81	1.54 ± 0.12	1.27 - 1.82
**Weight (kg)**	68.3 ± 19.9	35.1 - 137.0	68.5 ± 22.0	33.9 - 151.0
**BMI (kg/m**^**2**^**)**	28.2 ± 4.9	19.1 - 42.8	28.3 ± 5.6	19.6 - 47.4
**BMI Z Score**	1.8 ± 0.5	0.7 - 3.0	1.8 ± 0.6	0.5 - 3.2
**TBF**_**DEXA**_ **(%)**	44.4 ± 6.5*	28.4 - 59.7*	47.5 ± 6.7	29.6 - 63.1
**TBF**_**BIA Tanita® BC-532**_ **(%)**	34.5 ± 7.7*	23.4 – 58*	38.1 ± 6.6	26 - 59.2
	**N**	**Percentage**	**N**	**Percentage**
**Overweight (N, %)**	28	30.1	39	37.5
**Obesity (N, %)**	65	69.9	65	62.5

TBF DEXA (%): total body fat percentage assessed with dual-energy x-ray absorptiometry

TBF BIA Tanita® BC-532 (%): total body fat percentage assessed with the bioelectrical impedance analyser

Min: minimal value. Max: maximal value. SD: standard deviation

*p <  10^− 4^ (Student’s t-tests for paired samples)

One girl had a maximal value of 59.2% of TBF (%) by the BIA Tanita® BC-532. She was 14 years old, had a height of 1.72 m, a weight of 140.3 kg and a TBF DEXA (%) of 63.1%.

Two boys had a maximal value of 58% of TBF (%) by the BIA Tanita® BC-532. They were respectively 11 and 16 years old, had a height of 1.56 m and 1.79 m, a weight of 101 kg and 137 kg and a TBF DEXA (%) of 57.1 and 49.2%. The means of TBF (%) by the BIA Tanita® BC-532 were significantly different according to the Tanner stage only in girls (Table 2).

**Table 2:** Body fat percentage by the Tanita® BC-532, according to the Tanner stage

Tanner stage	I N (%)	IIN (%)	III N (%)	IV N (%)	V N (%)	***P***-Value
**Boys (*N =*** **93)**	42 (45.2%)	28 (30.1%)	7 (7.5%)	10 (10.8%)	6 (6.5%)	0.607
**TBF**_**BIA Tanita® BC-532**_ **(%)**	24.9 ± 7.1	23.4 ± 8.2	27.1 ± 4.6	26.9 ± 7.2	24.2 ± 12.1	
**Girls (*N =*** **104)**	23 (22.1%)	28 (26.9%)	15 (14.4%)	9 (8.7%)	29 (27.9%)	< 10^−4^
**TBF**_**BIA Tanita® BC-532**_ **(%)**	26.8 ± 4.0	29.5 ± 5.8	26.0 ± 7.4	33.2 ± 7.3	31.5 ± 6.2	

TBF BIA Tanita® BC-532 (%): total body fat percentage assessed with the bioelectrical impedance analyser, expressed as mean ± standard deviation (SD)

*p <  10^− 4^ (ANOVA)

The bivariate regression analyses showed that the TBF% _BIA Tanita® BC-532_ evaluated with the automated bioimpedance analyser was significantly correlated with the total body fat percentage measured with the DEXA (TBF% _DEXA_) in boys (r _Pearso*n*_ *=* 0.617) and girls (r _Pearson_ = 0.648) (*p* <  10^− 4^) (Table 3). Tanner stage was included in the bivariate regression in girls because of the significant differences observed in the means of TBF (%) by the BIA Tanita® BC-532, according to the Tanner stage in girls (Table 3). However, Tanner stage did not significantly contributed to improve the prediction of TBF% _DEXA_ by TBF% _BIA Tanita® BC-532_ in girls as shown in Table 3. Indeed, in the model adjusted on Tanner stage in girls, the variance explained was the same as in the model without adjustment (r _Pearson_: 0.648; R^2^ = 0.420; SEE = 5.18%; *p* <  10^− 4^). Tanner stage did not significantly contributed to the variance explanation in the model (r _Pearson_ partial: − 0.019; *P*-Value partial: 0.848) (Table 3).

**Table 3:** Univariate regression analysis between the percentage of total body fat assessed by the automated bioelectrical impedancemeter Tanita® BC-532 and measured by the dual-energy x-ray absorptiometry

		Model				TBF _**BIA Tanita®**_		Tanner stage	
		r _**Pearson**_	R^**2**^	SEE (%)	P-Value	r _**Pearson**_partial	P-Valuepartial	r _**Pearson**_partial	***P***-Valuepartial
**Boys (*N =*** **93)**	TBF _DEXA_ (%) = [0.617 × TBF _BIA Tanita® BC-532_ (%)] + 26.24	0.617	0.381	5.18	< 10^−4^	0.617	< 10^− 4^	–	–
**Girls (*N =*** **104)**	TBF _DEXA_ (%) = [0.648 × TBF _BIA Tanita® BC-532_ (%)] + 22.17	0.648	0.420	5.16	< 10^− 4^	0.648	< 10^− 4^	–	–
**Girls (*N =*** **104)**	Model adjusted on Tanner stage in Girls	0.648	0.420	5.18	< 10^−4^	0.618	< 10^−4^	−0.019	0.848

TBF DEXA (%): total body fat percentage assessed with dual-energy x-ray absorptiometry

TBF BIA Tanita® BC-532 (%): total body fat percentage assessed with the bioelectrical impedance analyser

**Fig. 2:**
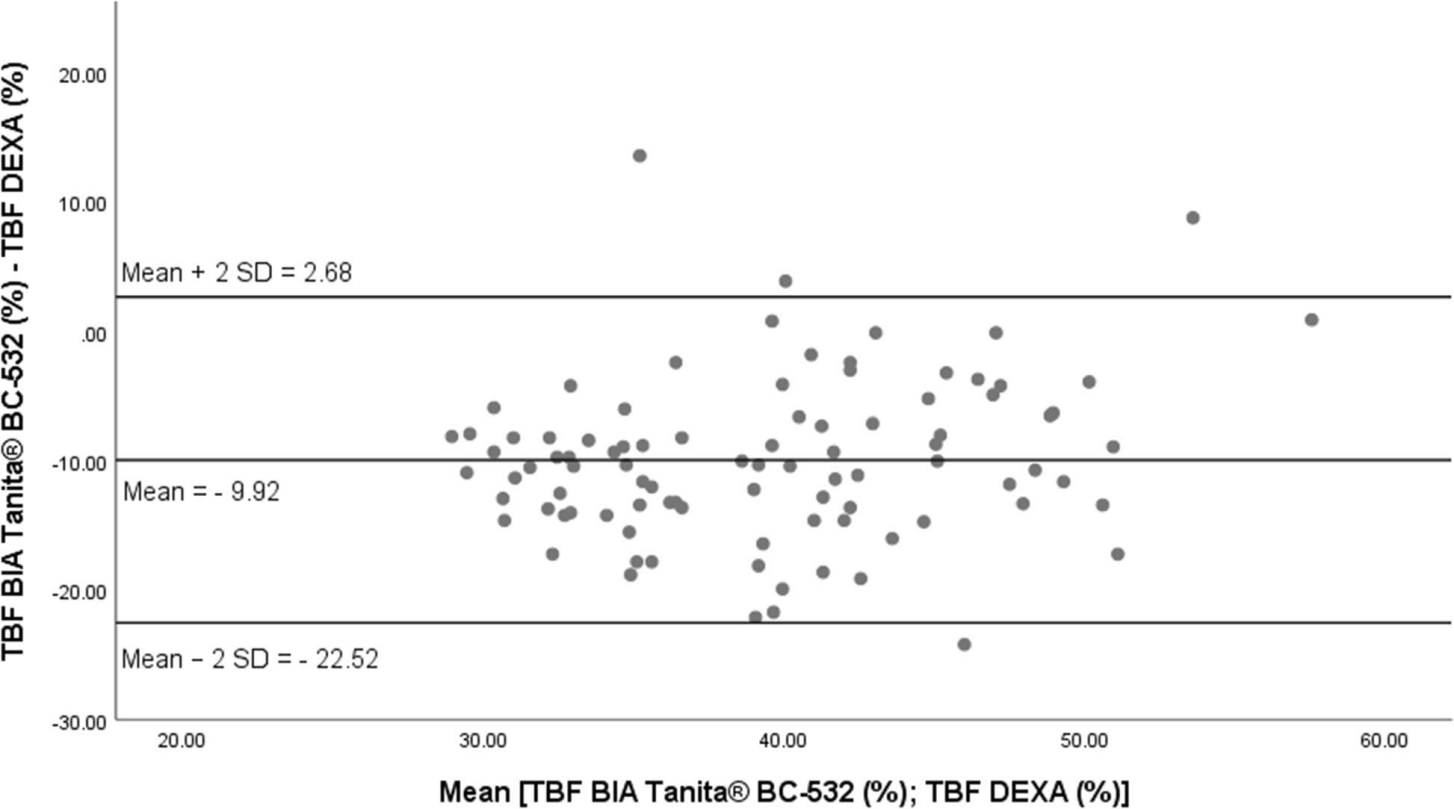
Bland and Altman representation of the differences between the percentages of total body fat assessed by Tanita® BC-532 and DEXA (Hologic® QDR4500W) in boys of White European ethnicity (*N =* 93)

**Fig. 3:**
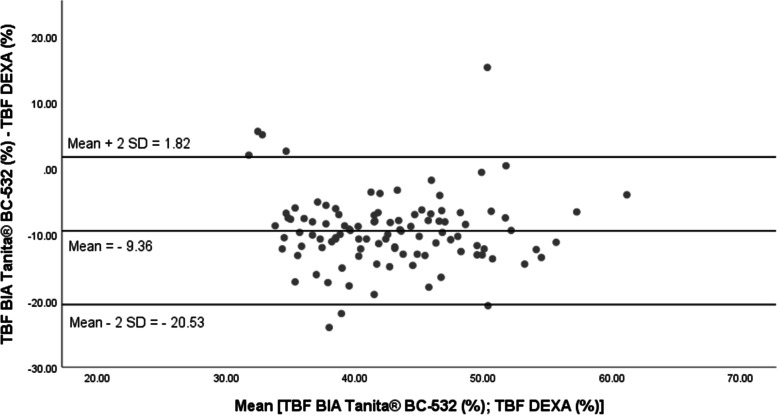
Bland and Altman representation of the differences between the total body fat percentages assessed by Tanita® BC-532 and DEXA (Hologic® QDR4500W) in girls of White European ethnicity (*N =* 104)

The standard error of estimation (SEE) of the TBF% by the BIA Tanita® BC-532 was about 5.18% in boys and 5.16% in girls (Table 3). In addition, the residual mean (absolute value) between the assessment of TBF% by the BIA Tanita® BC-532 and its measurement by DEXA [TBF _BIA Tanita® BC-532_ (%)-TBF _DEXA_ (%)] was extremely high (mean ± standard deviation = 10.52% ± 5.22% in boys, respectively 9.96% ± 4.40% in girls). The maximal absolute residual value was also very high, about 24% in both genders (Table 4). The adapted Bland and Altman representations show the differences between the total body fat percentage as evaluated by bioimpedance (TBF _BIA Tanita® BC-532_%) and measured by dual-energy x-ray absorptiometry (TBF _DEXA_ %) regressed across the average of TBF % as assessed by the 2 methods (1/2 TBF _BIA Tanita® BC-532_% + TBF _DEXA_ %) (Figs. 2 and 3). For both genders, the mean values of the (TBF _BIA Tanita® BC-532_% - TBF _DEXA_ %) residuals were different from zero, possibly implying the existence of a systematic error of the total body fat prediction with the Tanita® BC-532 (Residual mean: − 9.92% in boys; − 9.36% in girls). However, the differences between the TBF _BIA Tanita® BC-532_ (%) and the TBF _DEXA_ % values were not significantly correlated with the average of the two methods (Figs. 2 and 3).

**Table 4:** Evaluation of the precision of the automated bioelectrical impedancemeter Tanita® BC-532 to assess the total body fat, compared to the dual-energy x-ray absorptiometry: residual values

	Boys (***N =*** 93) TBF _DEXA_ (%) = [0.617 × TBF _BIA Tanita® BC-532_ (%)] + 26.24	Girls (***N =*** 104) TBF _DEXA_ (%) = [0.648 × TBF _BIA Tanita® BC-532_ (%)] + 22.17
**Residuals (%)**		
**Mean ± SD**	− 9.92 ± 6.30	−9.36 ± 5.59
**Min**	−24.20	− 24.00
**Max**	13.60	15.40
**Residuals (absolute value)**		
**Mean ± SD**	10.52 ± 5.22	9.96 ± 4.40
**Max**	24.20	24

TBF DEXA (%): total body fat percentage assessed with dual-energy x-ray absorptiometry

TBF BIA Tanita® BC-532 (%): total body fat percentage assessed with the bioelectrical impedance analyser Tanita® BC-532

Residuals (%) = [TBF BIA _BIA Tanita® BC-532_ (%)-TBF DEXA (%)]

## Discussion

The foot-to-foot automated bioimpedance anaysis devices [18–22, 30, 43–47] enable an easy and quick assessment of the total body fat, compared to the multi-frequencies BIA devices [48, 49] that have been developed and validated in specific populations and used in specialised areas. The positioning of the electrodes is easiest. Their low cost and the automated calculation of the total body fat facilitated their use by non specialised healthcare providers and researchers in body composition, as well as by the general public, without selecting population-specific body fat equations [30, 43–47].

We assessed the accuracy of the automated BIA device Tanita® BC-532 in comparison with the DEXA, gold standard measurement of total body fat. Several authors used the DEXA technique in order to investigate the accuracy of the *bioelectrical impedance* in predicting total fat mass [18–20, 23, 43, 45–47].

Our results showed that the values of TBF _DEXA_% were significantly different from the values of TBF _BIA Tanita® BC-532_% in both genders. This is in agreement with the findings of previously published studies showing significant differences in the TBF values evaluated by automated BIA devices and the DEXA [45–47]. In a study conducted in France by Lazzer et al. [45] in 53 adolescents with overweight or obesity, the two foot-to-foot BIA devices Tanita® BF-625 and Téfal BodymasterVision® showed low means differences between the TBF% assessed by BIA and DEXA. Similar results were observed in the study conducted by Kasvis et al. [47] using the automated BIA device Tanita® TBF-310 in 7-13 years old youth with overweight or obesity. In the study conducted by Barreira in 5–18 years old youth, the Tanita® SC-240 body composition analyser provided a different body fat estimation from the DEXA in boys and girls of White American ethnicity, unlike in boys and girls of Black American ethnicity [46].

The correlation coefficients and standard error of estimation between the TBF% _DEXA_ and the TBF% _BIA Tanita® BC-532_ are relatively high in our study and comparable to the values published in the litterature for other foot-to-foot automated bioimpedance anaysis devices such as the Tanita® BF-625, the Téfal BodymasterVision®, the Tanita® TBF-310 [45, 47]. However, compared to the DEXA, the absolute value of the residual mean between the assessment of the TBF% by the BIA Tanita® BC-532 and its measurement by DEXA was extremely high in our study, about 10.52% in boys, and 9.96% in girls, with a maximal value about 24%, namely ¼ of the total body fat. Previously published studies observed similar errors and limitations of the automated BIA devices in predicting total fat mass in both adults [18–20, 23] and youth [43, 45–47]. In children and adolescents, the difference between TBF assessed by BIA and measured by a gold standard fat mass method varied between − 12.3 and 13.7% [43]. Lazzer et al. [45] showed that the two foot-to-foot BIA devices Tanita® BF-625 and Téfal BodymasterVision® provided significantly different values of TBF% from DEXA. The average differences between body fat assessed by BIA and DEXA were about − 2.5% [TBF _DEXA_% - TBF _Tanita® BF-625_%] (*p* = 0.001), respectively − 1.8% [TBF _DEXA_% - TBF _Téfal BodymasterVision®_%] (*p* = 0.096) [45]. In addition, the two foot-to-foot BIA devices underestimated the TBF%, compared to the DEXA [45]. In our study, residuals (absolute value, mean ± SD) were about 10.52% ± 5.22% in boys and 9.96% ± 4.40% in girls. The Bland and Altman representations did not show any systematic under- or overestimation of the TBF% by the BIA Tanita® BC-532. In 7-13 years old youth from Montréal, Québec, Canada, the Bland and Altman representations showed a low level of agreement between the total body fat measured by DEXA and predicted by the BIA device Tanita® TBF-310 [47]. The average error between the measurement of body fat with DEXA and its estimation with the Tanita® SC-240 was about − 1.0% in a group of 5–18 years old youth of Black and White American ethnicity, the average absolute error was about 3.9% [46], although the BIA under-estimated the %TBF in the youth of White American ethnicity [46].

To the best of our knowledge, this is the first study evaluating the accuracy of the automated BIA device Tanita® BC-532 in youth. Previous validations of the Tanita® BC-532 by means of the DEXA were conducted in adults. Compared to the DEXA, the Tanita® BC-532 significantly underestimated the TBF% by 6.2 to 10.7% in 18–80 years old adults with a wide range of BMI from Shanghai [50].

These errors might be explained by the fact that the equations predicting the TBF% included in the BIA were developed in a population different from the study population where the BIA is used [30]. The performance of a predictive equation might vary according to the population, as previously shown in a multicentric study conducted with the hand-to-hand automated BIA device, OMRON® BF 306, in different populations from Netherlands, Italy and Finland [51]. The BIA devices are dedicated to be used within the specific populations where the predictive models of TBF were developed, and are not useful for other individuals and groups [27–30]. This might be a limitation of our study.

An additional limitation of the study is the difficulty to assess the hydration status especially as the automated BIA devices do not enable this assessment, although the girls took part to the study outside their menstrual period, which might have controlled, at least for a part, the consistency of the hydration status of the study participants.

Due to the high estimation errors of the total body fat by the automated BIA, it is important to carefully interpret the values of TBF obtained from automated BIA devices in the clinical setting, in particular when the values are used in order to diagnose obesity [30, 52].

Furthermore, when taking body composition measurements in young people, the assessment of puberty is important, although the epidemiological studies previously published on the relationship between obesity and puberty are controverted, especially in boys [53–56]. From one side, the earlier onset of puberty seems to be associated with obesity in girls, but not in boys having obesity who seems to have a late onset of puberty, while boys with overweight display an earlier puberty onset [53–56]. This controversy might be explained by a possible important limitation of the epidemiological studies to accurately assess the pubertal stages, in particular regarding the difficulty to differentiate between adipose tissue and thelarche in girls having obesity; and the difficulty to perform a physical examination and orchidometry in boys, which limits the examination to a subjective visual process, as underlined by Reinehr and Roth (2019) [53]. In our study, the means of TBF (%) by the BIA Tanita® BC-532 were significantly different according to the Tanner stage in girls. However, Tanner stage did not significantly contributed to improve the prediction of TBF% _DEXA_ by TBF% _BIA Tanita® BC-532_ in girls.

In addition, usually the algorithms used to calculate TBF values, and included in the BIA devices available in the market, are non-public. Therefore, we do not know whether these body fat predictive algorithms were adjusted on the puberty’s status. This in particular the case of the Tanita® BC-532 BIA device we used in the present work.

A limitation of the total body fat prediction by BIA lies in the fact that the method is based on a two exclusive body compartments model (fat mass and fat free mass) [15–17], which assimilate the water conducting the electric current to the whole fat free mass, without taking specifically in consideration the difference in the fat free mass component (bone, muscle and water compartments).

While affordable BIA technologies have improved over the years, they are not yet validated in all populations, including children, as our study highlights. There is a need for automated, validated, and affordable TBF measurement methods applicable to different populations. Such devices should safely and easily be used for clinical research and epidemiologic purposes but also in healthcare settings and to be used directly by individuals from home. Improving the equations/algorithms used to calculate TBF values in different populations is required. Such improvement could be done by combining for instance BIA data and anthropometric measurements of the human body. To include populations ranging from children to the elderly, such body dimensions should also be precise, validated, automated as well as affordable. Fully validated and automated devices for TBF measurements would help better diagnose patients with obesity but would also be unique in the digital health devices market, especially for the smart scales segment which is expected to show an annual growth rate (CAGR 2022-2026) of 7.27% worldwide and a projected market volume of US$4770.33 m by 2026 [57].

## Conclusions

The automated BIA device Tanita® BC-532 appears to be not accurate to assess total body fat in youth of White European ethnicity with overweight or obesity. There is a need to calibrate the BIA device before its use in the populations where it was not previously validated.

## Data Availability

The data that support the findings of this study are available from the Luxembourg Institute of Health but restrictions apply to the availability of these data, and so are not publicly available. Data are however available from the corresponding author upon reasonable request and with permission of the Luxembourg Institute of Health.

## Abbreviations

BMI: Body mass index
CAGR: Compound Annual Growth Rate
DEXA: Dual-energy x-ray absorptiometry
kHz: Kilohertz
mA: Milliampere
SEE: Standard error of estimation
SD: Standard deviation
TBF: Total body fat
TBF%: Total body fat percentage
WHO: World Health Organisation

## References

[1] WHO: Obesity: preventing and managing the global epidemic. Report of a WHO Consultation. WHO Technical Report Series 894: 2000; 252. 11234459

[2] Lambert BS, Oliver JM, Katts GR, Green JS, Martin SE, Crouse SF (2012). DEXA or BMI: clinical considerations for evaluating obesity in collegiate division I-A American football athletes. Clin J Sport Med.

[3] Okorodudu DO, Jumean MF, Montori VM, Romero-Corral A, Somers VK, Erwin PJ, Lopez-Jimenez F (2010). Diagnostic performance of body mass index to identify obesity as defined by body adiposity: a systematic review and meta-analysis. Int J Obes.

[4] Piers LS, Soares MJ, Frandsen SL, O'Dea K (2000). Indirect estimates of body composition are useful for groups but unreliable in individuals. Int J Obes Relat Metab Disord.

[5] Wellens RI, Roche AF, Khamis HJ, Jackson AS, Pollock ML, Siervogel RM (1996). Relationships between the body mass index and body composition. Obes Res.

[6] Garn SM, Leonard WR, Hawthorne VM (1986). Three limitations of the body mass index. Am J Clin Nutr.

[7] Adab P, Pallan M, Whincup P. Is BMI the best measure of obesity? Bmj. 2018;360(k1274). 10.1136/bmj.k1274 29599212

[8] Frankenfield DC, Rowe WA, Cooney RN, Smith JS, Becker D (2001). Limits of body mass index to detect obesity and predict body composition. Nutrition.

[9] Albanese CV, Diessel E, Genant HK (2003). Clinical applications of body composition measurements using DXA. J Clin Densitom.

[10] Pietrobelli A, Formica C, Wang Z, Heymsfield SB (1996). Dual-energy X-ray absorptiometry body composition model: review of physical concepts. Am J Phys.

[11] Jensen MD, Kanaley JA, Roust LR, O'Brien PC, Braun JS, Dunn WL, Wahner HW (1993). Assessment of body composition with use of dual-energy x-ray absorptiometry: evaluation and comparison with other methods. Mayo Clin Proc.

[12] Pritchard JE, Nowson CA, Strauss BJ, Carlson JS, Kaymakci B, Wark JD (1993). Evaluation of dual energy X-ray absorptiometry as a method of measurement of body fat. Eur J Clin Nutr.

[13] Moreira OC, Oliveira CEP, De Paz JA (2018). Dual energy X-ray absorptiometry (DXA) reliability and intraobserver reproducibility for segmental body composition measuring. Nutr Hosp.

[14] Simpson JA, Lobo DN, Anderson JA, Macdonald IA, Perkins AC, Neal KR, Allison SP, Rowlands BJ (2001). Body water compartment measurements: a comparison of bioelectrical impedance analysis with tritium and sodium bromide dilution techniques. Clin Nutr.

[15] Houtkooper LB, Lohman TG, Going SB, Howell WH (1996). Why bioelectrical impedance analysis should be used for estimating adiposity. Am J Clin Nutr.

[16] Wang J, Wang X (2003). Prediction formulas for estimating body fat percent of obesity from bioelectrical impedance. Wei Sheng Yan Jiu.

[17] Lukaski HC, Bolonchuk WW, Siders WA, Hall CB (1990). Body composition assessment of athletes using bioelectrical impedance measurements. J Sports Med Phys Fitness.

[18] Lloret Linares C, Ciangura C, Bouillot JL, Coupaye M, Decleves X, Poitou C, Basdevant A, Oppert JM (2011). Validity of leg-to-leg bioelectrical impedance analysis to estimate body fat in obesity. Obes Surg.

[19] Dixon JB, Bhasker AG, Lambert GW, Lakdawala M (2016). Leg to leg bioelectrical impedance analysis of percentage fat mass in obese patients-can it tell us more than we already know?. Surg Obes Relat Dis.

[20] Rockamann RA, Dalton EK, Arabas JL, Jorn L, Mayhew JL (2017). Validity of arm-to-arm BIA devices compared to DXA for estimating % fat in college men and women. Int J Exerc Sci.

[21] Gibson AL, Heyward VH, Mermier CM (2000). Predictive accuracy of Omron body logic analyzer in estimating relative body fat of adults. Int J Sport Nutr Exerc Metab.

[22] Weaver AM, Hill AC, Andreacci JL, Dixon CB (2009). Evaluation of hand-to-hand bioelectrical impedance analysis for estimating percent body fat in young adults. Int J Exerc Sci.

[23] Mally K, Trentmann J, Heller M, Dittmar M (2011). Reliability and accuracy of segmental bioelectrical impedance analysis for assessing muscle and fat mass in older Europeans: a comparison with dual-energy X-ray absorptiometry. Eur J Appl Physiol.

[24] Meyer KA, Friend S, Hannan PJ, Himes JH, Demerath EW, Neumark-Sztainer D (2011). Ethnic variation in body composition assessment in a sample of adolescent girls. Int J Pediatr Obes.

[25] Delvaux I, Van Cauwenberghe J, Den Hond E, Schoeters G, Govarts E, Nelen V, Baeyens W, Van Larebeke N, Sioen I (2014). Prenatal exposure to environmental contaminants and body composition at age 7-9 years. Environ Res.

[26] Xu F, Greene GW, Earp JE, Adami A, Delmonico MJ, Lofgren IE, Greaney ML (2020). Relationships of physical activity and diet quality with body composition and fat distribution in US adults. Obesity (Silver Spring).

[27] Khalil SF, Mohktar MS, Ibrahim F (2014). The theory and fundamentals of bioimpedance analysis in clinical status monitoring and diagnosis of diseases. Sensors (Basel).

[28] Deurenberg P, Deurenberg-Yap M, Schouten FJ (2002). Validity of total and segmental impedance measurements for prediction of body composition across ethnic population groups. Eur J Clin Nutr.

[29] Deurenberg P, Deurenberg-Yap M (2003). Validity of body composition methods across ethnic population groups. Acta Diabetol.

[30] Orsso CE, Gonzalez MC, Maisch MJ, Haqq AM, Prado CM. Using bioelectrical impedance analysis in children and adolescents: pressing issues. Eur J Clin Nutr. 2021. 10.1038/s41430-021-01018-w 34620999

[31] Samouda H, de Beaufort C, Stranges S, Guinhouya BC, Gilson G, Hirsch M, Jacobs J, Leite S, Vaillant M, Dadoun F (2015). Adding anthropometric measures of regional adiposity to BMI improves prediction of cardiometabolic, inflammatory and adipokines profiles in youths: a cross-sectional study. BMC Pediatr.

[32] Samouda H, De Beaufort C, Stranges S, Hirsch M, Van Nieuwenhuyse JP, Dooms G, Gilson G, Keunen O, Leite S, Vaillant M (2016). Cardiometabolic risk: leg fat is protective during childhood. Pediatr Diabetes.

[33] Samouda H, De Beaufort C, Stranges S, Van Nieuwenhuyse JP, Dooms G, Keunen O, Leite S, Vaillant M, Lair ML, Dadoun F (2017). Subtraction of subcutaneous fat to improve the prediction of visceral adiposity: exploring a new anthropometric track in overweight and obese youth. Pediatr Diabetes.

[34] Samouda H, De Beaufort C, Gilson G, Schritz A, Vaillant M, Ghaddhab C, Ruiz-Castell M, Huiart L, Dohet F, Weber B (2020). Relationship of oxidative stress to visceral adiposity in youth and role played by vitamin D. Pediatr Diabetes.

[35] Cole TJ, Bellizzi MC, Flegal KM, Dietz WH (2000). Establishing a standard definition for child overweight and obesity worldwide: international survey. BMJ.

[36] Lohmann TG, Roche AF, Martorell R (1988). Anthropometric standardization reference manual.

[37] Fredriks AM, van Buuren S, Wit JM, Verloove-Vanhorick SP (2000). Body index measurements in 1996-7 compared with 1980. Arch Dis Child.

[38] Software for LMS method. http://homepagemaccom/tjcole/FileSharing1html. Accessed December 26, 2021.

[39] Marshall WA, Tanner JM (1969). Variations in pattern of pubertal changes in girls. Arch Dis Child.

[40] Marshall WA, Tanner JM (1970). Variations in the pattern of pubertal changes in boys. Arch Dis Child.

[41] Samouda H, Dutour A, Chaumoitre K, Panuel M, Dutour O, Dadoun F (2013). VAT=TAAT-SAAT: innovative anthropometric model to predict visceral adipose tissue without resort to CT-scan or DXA. Obesity (Silver Spring).

[42] Bland JM, Altman DG (1999). Measuring agreement in method comparison studies. Stat Methods Med Res.

[43] Talma H, Chinapaw MJ, Bakker B, HiraSing RA, Terwee CB, Altenburg TM (2013). Bioelectrical impedance analysis to estimate body composition in children and adolescents: a systematic review and evidence appraisal of validity, responsiveness, reliability and measurement error. Obes Rev.

[44] Brantlov S, Jodal L, Lange A, Rittig S, Ward LC (2017). Standardisation of bioelectrical impedance analysis for the estimation of body composition in healthy paediatric populations: a systematic review. J Med Eng Technol.

[45] Lazzer S, Boirie Y, Meyer M, Vermorel M (2003). Evaluation of two foot-to-foot bioelectrical impedance analysers to assess body composition in overweight and obese adolescents. Br J Nutr.

[46] Barreira TV, Staiano AE, Katzmarzyk PT (2013). Validity assessment of a portable bioimpedance scale to estimate body fat percentage in white and African-American children and adolescents. Pediatr Obes.

[47] Kasvis P, Cohen TR, Loiselle SE, Kim N, Hazell TJ, Vanstone CA, Rodd C, Plourde H, Weiler HA (2015). Foot-to-foot bioelectrical impedance accurately tracks direction of adiposity change in overweight and obese 7- to 13-year-old children. Nutr Res.

[48] Kushner RF (1992). Bioelectrical impedance analysis: a review of principles and applications. J Am Coll Nutr.

[49] Xitron: HYDRA ECF/ICF Model 4200). Bioimpedance spectrum analyzer. For measuring intracellular and extracellular fluid volumes. In: Operating Manual Revision 103. San Diego, CA, USA: XITRON, TECHNOLOGIES; INC.; 2007.

[50] Wang JG, Zhang Y, Chen HE, Li Y, Cheng XG, Xu L, Guo Z, Zhao XS, Sato T, Cao QY (2013). Comparison of two bioelectrical impedance analysis devices with dual energy X-ray absorptiometry and magnetic resonance imaging in the estimation of body composition. J Strength Cond Res.

[51] Deurenberg P, Andreoli A, Borg P, Kukkonen-Harjula K, de Lorenzo A, van Marken Lichtenbelt WD, Testolin G, Vigano R, Vollaard N (2001). The validity of predicted body fat percentage from body mass index and from impedance in samples of five European populations. Eur J Clin Nutr.

[52] Marra M, Sammarco R, De Lorenzo A, Iellamo F, Siervo M, Pietrobelli A, Donini LM, Santarpia L, Cataldi M, Pasanisi F (2019). Assessment of body composition in health and disease using bioelectrical impedance analysis (BIA) and dual energy X-ray absorptiometry (DXA): a critical overview. Contrast Media Mol Imaging.

[53] Reinehr T, Roth CL (2019). Is there a causal relationship between obesity and puberty?. Lancet Child Adolesc Health.

[54] Crocker MK, Stern EA, Sedaka NM, Shomaker LB, Brady SM, Ali AH, Shawker TH, Hubbard VS, Yanovski JA (2014). Sexual dimorphisms in the associations of BMI and body fat with indices of pubertal development in girls and boys. J Clin Endocrinol Metab.

[55] Biro FM, Khoury P, Morrison JA (2006). Influence of obesity on timing of puberty. Int J Androl.

[56] Wang Y (2002). Is obesity associated with early sexual maturation? A comparison of the association in American boys versus girls. Pediatrics.

[57] Smart Scales - Worldwide. Accessed February 16, 2022. https://www.statista.com/outlook/dmo/digital-health/digital-fitness-well-being/digital-fitness-well-being-devices/smart-scales/worldwide

